# UWB Wind Turbine Blade Deflection Sensing for Wind Energy Cost Reduction

**DOI:** 10.3390/s150819768

**Published:** 2015-08-12

**Authors:** Shuai Zhang, Tobias Lindstrøm Jensen, Ondrej Franek, Patrick C. F. Eggers, Kim Olesen, Claus Byskov, Gert Frølund Pedersen

**Affiliations:** 1 Antennas, Propagation and Radio Networking section at the Department of Electronic Systems, Faculty of Engineering and Science, Aalborg University, Aalborg 9100, Denmark; E-Mails: of@es.aau.dk (O.F.); pe@es.aau.dk (P.C.F.E.); ko@es.aau.dk (K.O.); gfp@es.aau.dk (G.F.P.); 2 Signal and Information Processing section at the Department of Electronic Systems, Faculty of Engineering and Science, Aalborg University, Aalborg 9100, Denmark; E-Mail: tlj@es.aau.dk; 3 LM Wind Power, Jupitervej 6, Kolding DK-6000, Denmark; E-Mail: clby@lmwindpower.com

**Keywords:** UWB, radio link, wind turbine, blade deflection

## Abstract

A new application of utilizing ultra-wideband (UWB) technology to sense wind turbine blade deflections is introduced in this paper for wind energy cost reduction. The lower UWB band of 3.1–5.3 GHz is applied. On each blade, there will be one UWB blade deflection sensing system, which consists of two UWB antennas at the blade root and one UWB antenna at the blade tip. The detailed topology and challenges of this deflection sensing system are addressed. Due to the complexity of the problem, this paper will first realize the on-blade UWB radio link in the simplest case, where the tip antenna is situated outside (and on the surface of) a blade tip. To investigate this case, full-blade time-domain measurements are designed and conducted under different deflections. The detailed measurement setups and results are provided. If the root and tip antenna locations are properly selected, the first pulse is always of sufficient quality for accurate estimations under different deflections. The measured results reveal that the blade tip-root distance and blade deflection can be accurately estimated in the complicated and lossy wireless channels around a wind turbine blade. Some future research topics on this application are listed finally.

## 1. Introduction

**Figure 1:**
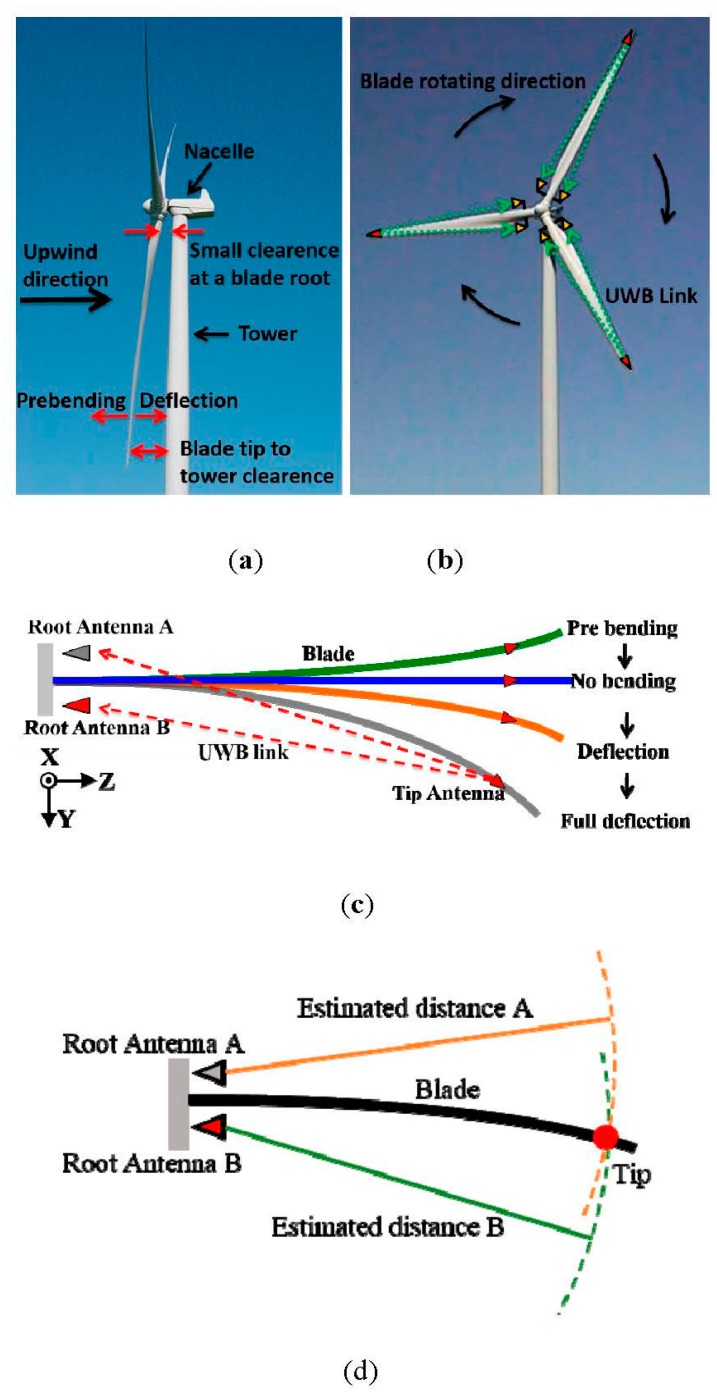
(**a**) The side view of a wind turbine; (**b**) the wind turbine with three UWB blade deflection sensing systems; (**c**) the UWB blade deflection sensing system topology and (**d**) a sketch of triangulation.

The number one priority along the entire value chain in the renewable energy industry is to reduce the cost per produced unit of electricity, the Cost of Energy (CoE). When the CoE of renewable energy sources is reduced below the CoE of fossil fuels, then our dependence of fossil fuels and subsidies for renewable energy sources can be eliminated and pave the way for a green and sustainable power production. For wind energy, the best method to reduce CoE is to employ longer blades. Wind farms around the globe are increasingly sited in regions with medium to low wind speeds. The need for increasing the area swept by the rotor, *i.e.*, the length of the blades, is thus reinforced. However, increasing the blade length comes at the expense of increasing mass of the blades. As a rule of thumb the mass of a wind turbine blade increases with the power of three to the length of the blade. The increased mass of the blades build stiffness into the blade such that the deflection of blades is kept within a design envelope. The design envelope states that the deflection of a fully loaded blade must not exceed a specified blade tip to tower clearance limit as shown in Figure 1a. This limit is typically defined as 70% of the blade tip to tower clearance of the unloaded blade. However, sensor technologies currently used in wind turbine blades lack capabilities to measure deflection reliably. If a technology could measure blade deflection on wind turbines in operation, in a reliable and cost efficient way, then blade structural designs, wind turbine controls and safety margins in combination could pave the way for use of larger blades and thus reduce the CoE.

Currently, most common used blade deflection seniors are the strain gauge, including optic Fiber Bragg Gratings (FBG) [1,2] technologies. The measurement of strain in blade material and the transformation of strain to loads and deflections are used only in prototype setups because of demanding calibration procedures. However, the calibration procedures cannot be cost-effectively carried out for a fleet of wind turbines. MEMS devices containing accelerometers and gyroscopes [3] have also been used in prototype setups only due to its vulnerability to lightning strikes. A single lightning strike in the neighborhood of the wind turbine frequently causes induced currents at levels that damage these devices. In [4,5], a laser has been installed within a tower. The blade deflection can be monitored every time the blade passes by the tower. However, it is difficult to utilize this technique to always sense the deflection when the blade runs around the clock. The alternative to sensor technology for a longer and lighter blade is use of advanced materials, *i.e.*, carbon/glass fiber hybrid materials [6].

Using radio technology to measure deflections of wind turbine blades has been described in patent applications by several of the largest players in the wind energy industry, but none of these applications have materialized into a technology that can actually be deployed into wind turbines in the field [7]. A key issue to the lacking practical use is the harsh environment in which the wind turbine blade operates. The tip of a modern on-shore wind turbine will at its top point reach 150–200 m above the ground, and travel with a velocity of 70–90 m/s close to constantly around the clock during each day of the year. The blade tip will attract lightning strikes, be subject to erosion of material on the leading edge of the blade, and even the slightest surface discontinuities will be a source of acoustic noise. The winning technology will take into account all these factors. Compared with all the available radio technologies, ultra-wideband (UWB) would be one of the most promising candidates for the accurate blade deflection sensing. UWB is a radio technology utilizing a very low power level and a large portion of radio spectrum. Due to the large bandwidth in frequency domain, UWB signal exhibits a narrow pulse in time domain. High-accuracy localization in centimeter level can be achieved by estimating the time-of-flight (TOA) of the first signal pulse. UWB localization technology has been widely used in, *i.e.*, sensor networks [8], Radio-frequency identification (RFID)-UWB systems [9], and UWB medical diagnostics [10]. However, it has not been attempted to apply UWB for blade deflection sensing.

In this paper, we will for the first time utilize UWB technology to sense wind turbine blade deflection for wind energy cost reduction. As a new application, the detailed topology and challenges (in antennas and propagations) of a blade deflection sensing system are introduced. This paper does not intend to solve all the challenges due to the complexity of the problem. The antenna designs will not be the scope of this paper. Instead, we will first focus on proposing a solution to the challenges of a UWB radio link and realizing the blade deflection sensing in the simplest case, where a tip antenna is placed outside (and on the surface of) a blade tip. To achieve this purpose, full-blade time-domain measurements will be proposed and conducted under different deflections.

## 2. Topology and Challenges of a Blade Deflection Sensing System

### 2.1. Topology of a Blade Deflection Sensing System

A conventional wind turbine has three blades. On each blade, there will be one UWB blade deflection sensing system, as illustrated in Figure 1b. Each sensing system consists of two UWB antennas at the root and one UWB antenna at the tip end. Two root antennas are fixed on the blade root and will rotate together with the blade on a real wind turbine. In this way, two root antennas and one tip antenna will always be in the same coordinate system. Please note that the root antennas cannot be in the stationary positions, *i.e.*, tower or nacelle (see Figure 1a), since it will be very difficult for the system to track the extremely fast moving blade tip. The distance between the root and the tip antennas is estimated by the TOA of the first UWB pulse rising edge. A triangulation will then be performed with the two estimated root-tip antenna distances to determine the location of the blade tip antenna (as well as the location of the blade tip), as shown in Figure 1d. A standing assumption is that the stiffness of the blade along the xz plane (see Figure 1b) makes the blade deflection restricted to the yz plane. In this way, the blade deflection is estimated with the measured results obtained by using the UWB technology.

In Figure 1c, the topology of a UWB blade deflection sensing system is demonstrated. In practice, an unloaded blade is designed to be bent to the -y direction in Figure 1c, which is called “pre bending”. When the wind loads on the blade, the wind turbine starts operating and the blade will become straight (no bending) or further deflected. Therefore, the UWB tip-root link needs to exhibit an unambiguous and predictable path (preferably line-of-sight (LOS)) at least from no bending to full deflection (see Figure 1c). This will facilitate the accurate estimation of the root-tip antenna distance and the blade deflection in the same deflection range.

The lower UWB band of 3.1–5.3 GHz is selected for this new application. Compared with the higher UWB band of 6–10.6 GHz, the RF components and pulse modules in the lower band are much cheaper, less lossy and commercially available. The emitted power of UWB signals is limited by the effective isotropic radiated power (EIRP) of −41.3 dBm/MHz [11]. In this system, one-way time-of-flight ranging is applied. One active radio is transmitted from the tip antenna to the two root antennas. The root antennas are only for receiving.

### 2.2. Challenges in Antennas and Propagations for a Blade Deflection Sensing System

It is very challenging to build useful UWB radio links and design antennas in order to realize this blade deflection sensing system. There are mainly two reasons. First, a conventional blade is approximately a hollow dielectric cylinder, which is made of long, massive and lossy glass fiber. The glass fiber has the dielectric permittivity of 4.4 and loss tangent of 0.025. The length of a blade, commonly used nowadays, is from 30 m to over 80 m. The thickness of a blade is changing gradually from around 5 cm at the tip to around 2 m at the root. The blade shell material thickness is changing gradually from around 7 mm at the tip end to several centimeters at the root. Second, there is very limited clearance between the blade root and tower, as illustrated in Figure 1a. Typically, this clearance is less than 1.5 m, which limits the maximal root antenna height off the blade root surface. Therefore, the UWB radio link will always be in a complicated lossy wireless environment closely around a wind turbine blade from pre bending to full deflection (see Figure 1c), especially when the blade is straight.

The challenges in the UWB radio links mainly focus on the following issues: (1) as mentioned above, the tip-root antenna distance is estimated by the TOA of the first UWB pulse rising edge. The first pulse will experience amplitude changes and shadowing with the blade deflecting. The signal amplitude changes need to be investigated and the signal shadowing has to be avoided; (2) Compared to the actual TOA, a time delay of the first pulse may occur since the electromagnetic radio is close to and may interact with the blade shell material. However, this delay should be a fixed delay in different deflections, which can be calibrated in practical applications; (3) Due to the massive lossy blade material, the first pulse amplitude will be attenuated and the pulse signal poses a problem getting out at a low angle along the blade; (4) The first pulse will suffer from strong interference and cancellation effects from the multipath environment, *i.e.*, reflections and surface waves. Successfully minimizing these interference and cancellation is important to accurately estimate the root-tip distance and the blade deflection.

In antenna designs, the challenges are in the tip end. In general, a tip antenna should cover the lower UWB band of 3.1–5.3 GHz with stable phase centers. Since the massive and lossy blade material is in the near field of the tip antenna, the near-field interactions between the blade and the tip antenna need to be considered. Moreover, the impacts of lightning protection on the antenna performance have to be taken into account. In addition, there are also some specific challenges depending on the tip antenna locations. If a tip antenna is placed outside a blade, it has to be designed as an extremely low-profile structure on the blade surface. Due to aerodynamics, any protrusion on the blade tip would increase air drag and cause unacceptable acoustic noise. The outside tip antenna should also have a high (endfire) gain towards the blade root direction. When a tip antenna is placed inside a blade, there is less limitation on the antenna volume than the outside antenna. However, the inside antenna should have a higher gain to compensate the penetration loss through the blade shell. Furthermore, the multipath effects become very severe with an inside tip antenna, which raise higher requirements on channel analyses.

Compared with the inside tip antenna case, the challenges in the UWB radio link with a tip antenna placed outside a blade are much smaller and the wireless channel is also much simpler. Therefore, this paper will first solve the UWB radio link challenges for the outside tip antenna case. The antenna designs will not be involved in this paper. In the following, we will design full-blade time-domain measurements to realize the UWB radio links and the blade deflection sensing with a tip antenna placed outside a blade. The proposed system will be evaluated in a noisy and lossy wireless environment.

## 3. Full-Blade Time-Domain Measurement Setups

### 3.1. Test System Setups

**Figure 2:**
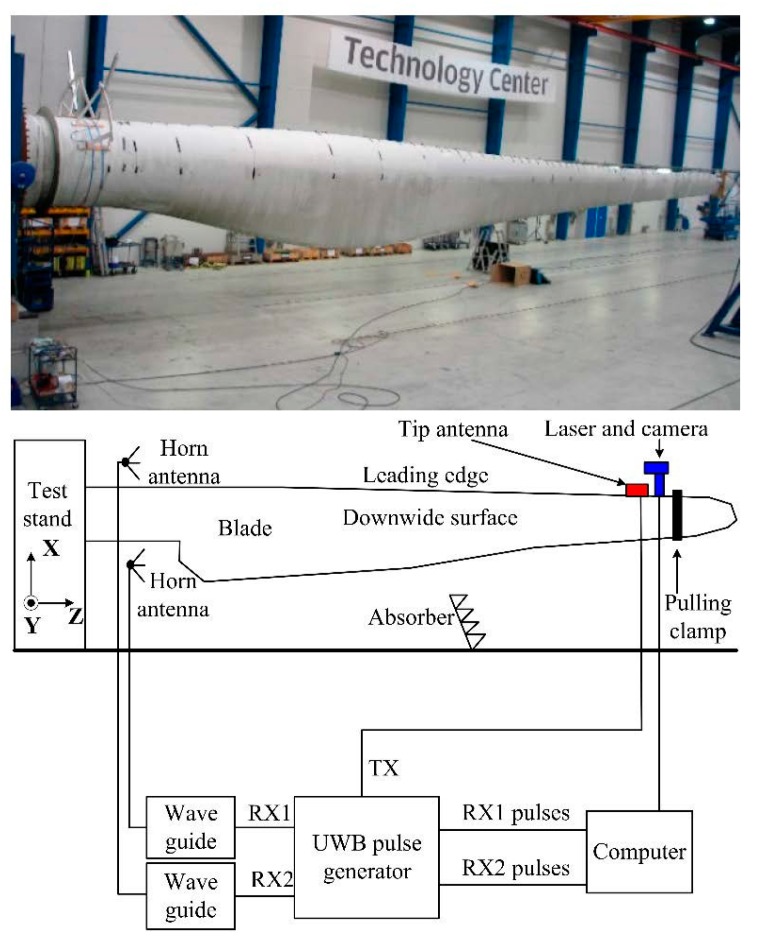
Test system setups.

The test system setups are shown in Figure 2. To validate the technology, a 37.3-m blade is mounted to a static test stand at LM wind power test center [12]. Controlled deflection of the blade is applied. A static pulling of the blade is performed with a single pulling clamp (see Figure 2) on the blade positioned 2.3 m from the blade tip. With this clamp position, the pulling can change the blade deflection along y axis in Figure 2, from the pre bending at −1.0 m to approximate 1.5 m in the deflection direction (or +y direction). Please note that, in practice, the blade can be pre-bent and fully deflected more than the possible values in the test center. However, for safety reasons, the deflection from about −1 m to 1.5 m is the maximal allowed range for this setup in the test center. In addition, some radio absorbers are placed on the floor to reduce multipath reflections from the concrete floor.

In real wind turbine applications, in order to limit service operations at the tip end, the powered components in the blade (see Figure 2) will be placed at the blade root end and the transmitter will be connected to the tip end via a long cable in the blade. Cables from the rotor hub feeds power to these components.

### 3.2. Root Antenna and Tip Antenna Locations

**Figure 3:**
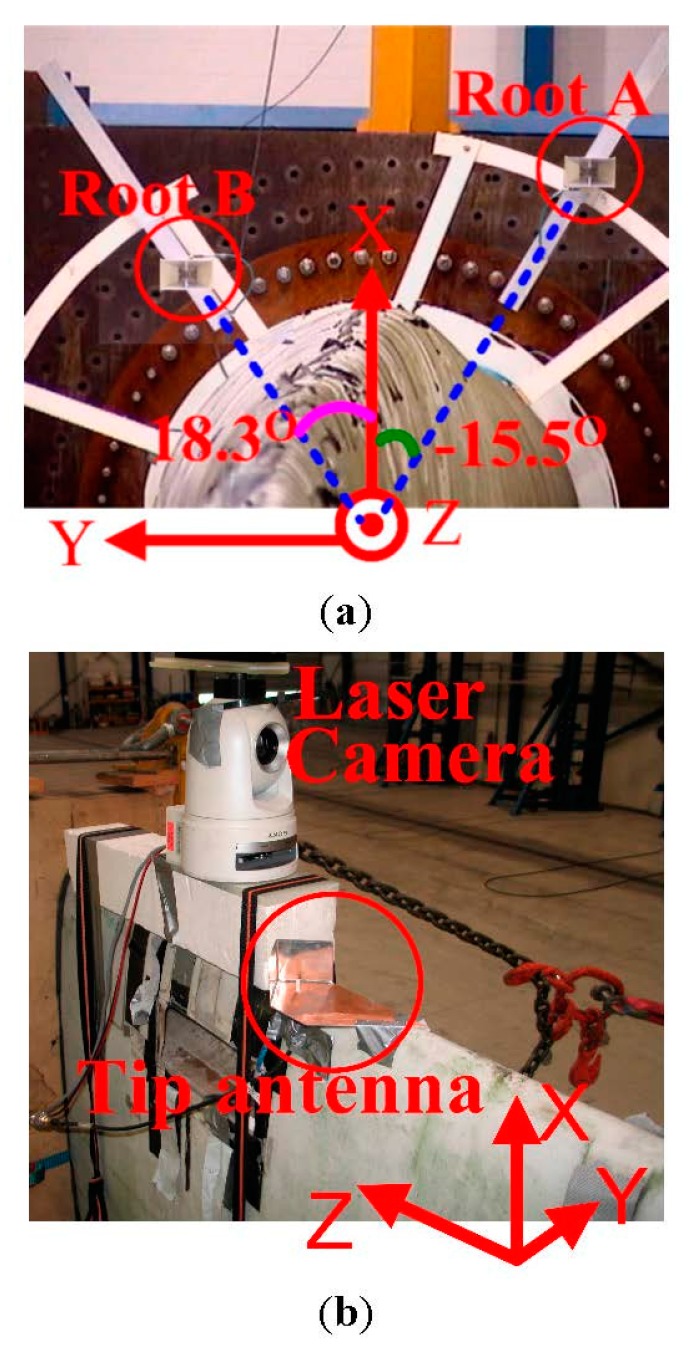
Locations of (**a**) root antennas and (**b**) tip antenna.

In this experiment, the locations of root antennas and tip antenna have to be optimized in order to solve the challenges in the UWB radio links mentioned above. The locations of the root antennas and tip antenna are illustrated in Figure 3a,b, respectively. Since the blade is over 37 m long and we need to achieve an accurate deflection sensing, it is critically important to measure the root antennas and tip antenna physical locations in the defined coordinates carefully in order to minimize the uncertainty in the measurement.

Two EMCO 3115 horn antennas [13] are used as the root antennas. In the band of 3.1–5.3 GHz, the horn antenna gain is from around 8.5 dBi to 11 dBi with stable phase centers. As demonstrated in Figure 3a, the root antenna A and the root antenna B are placed at the angles of −15.5° and 18.3°, with the heights of 1.07 m and 0.6 m off the blade root surface, respectively. The root antenna angles and heights have been carefully scanned and selected with the consideration of the following three factors: First, the two root antennas should be able to receive the LOS pulse components under different deflections, so that the first pulse will experience less amplitude changes and the shadowing can be avoided. If this requirement is satisfied, the LOS component can also be obtained in a real application. Second, the two root antennas should be separated in the y direction as much as possible to improve the triangulation accuracy. Finally, the first pulse will be severely attenuated and delayed due to the lossy blade material until the root antenna height off the blade surface reaches a certain value. As the root antenna height increases, the interference and cancellation effects from the multipath will also become less and less. In the meantime, the mechanical installing stability of the root antenna requires its height be as small as possible. Therefore, the minimum root antenna height should be applied, which can simultaneously achieve good first pulse qualities under different deflections and facilitate the mechanical stability.

The tip antenna is a corner reflected monopole (see Figure 3b), which has stable phase centers. It covers the band of 3.1–5.3 GHz with the gain of around 7 dBi towards -z direction. The tip antenna is positioned on the leading edge (see Figure 2) of the blade instead of the other places in order to make the UWB pulses experience as little lossy blade material as possible. In this way, the first pulse attenuation and delay can be minimized. In addition, the tip antenna is about 3 m from the blade tip (0.7 m from the pulling clamp), where the clamp will not interfere with the measurement (see Figure 2). During the measurements, the polarizations of the root and tip antennas are kept the same.

In addition, in the practical applications, the root antennas and tip antenna can be located in the same positions as those in this paper, if a tip antenna is placed on the surface of a blade tip.

### 3.3. UWB Pulse Generator and Signal Processing Algorithm

A UWB pulse generator is built (see Figure 2). This generator provides a Gaussian pulse signal with the RF transmissions from 3.1 GHz to 5.3 GHz. The signal sampling period is 6.1 × 10^−11^ s. The noise figure of the generator is 4.8 dB. In this pulse generator, there are two receiving ports and one transmitting port (see in Figure 2) connected to the two root antennas and one tip antenna, respectively. In addition, during the measurement two waveguides are used in front of the receiving ports of the pulse generator for the lightning protection in practical applications.

In [14], signal processing algorithms for a UWB blade deflection sensing system are investigated. The UWB signal processing algorithm in [14] will be applied for the recorded pulses. The algorithm is based on an approximate maximum *a posteriori* (MAP) estimator, which is exploiting the contextual prior information and a rising edge approximation. Conceptually, the algorithm tries to match the waveform of the received signal rising edge with a template pulse rising edge using the least-squares error criterion. Since the algorithm uses a rising edge approximation, it could reduce the impact of multipath environment and increase the estimation accuracy. The proposed algorithm in [14] is only a constant factor more computationally demanding than the correlation method, and can e.g., run in real-time on a standard laptop.

### 3.4. Laser Range Finder (Reference for Distance Estimation)

During different deflections, the distance between the tip antenna and each root antenna is measured by both laser and UWB methods simultaneously for comparison. A controlled laser measurement system of Leica D8 is utilized as the reference for the UWB distance estimation. The laser range finder is equipped with a camera (see Figure 3b) connected to a computer, which makes it possible from a computer to view the target of a laser beam and position this beam on the right location. The starting point of the laser mounted on the camera is positioned over the tip antenna. The accuracy of Leica D8 range finder is specified to ±0.15 mm/m [15], equivalent to ±5 mm over the distance of 33 m in the designed measurements. Therefore, the laser range finder can provide sufficient accuracy to be the reference for the distance estimation.

### 3.5. Electronic Wires (Reference for Deflection Tracking)

The laser range finder can measure the blade tip-root distance with a 5 mm tolerance. However, this 5 mm tolerance will be translated into a 50 mm error after the triangulation for the blade deflection estimation, which is not suitable as the reference for the UWB blade deflection tracking.

**Figure 4:**
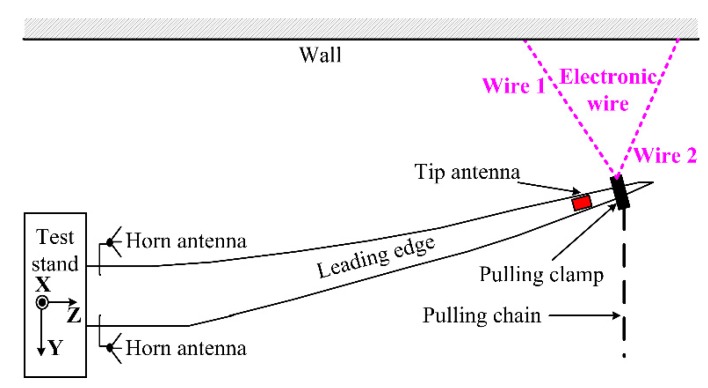
Electronic wire setups.

The deflection estimation from the triangulation of electronic wires are considered more accurate than the laser range finder. The measurement error of electronic wires after triangulation is less than 5 mm. The electronic wire is a wire which can precisely measure its physical length. The electronic wires are devices certified by an accredited institute. A string is pulled from the device and the length in meters of the pulled string is read out to an electronic interface. As illustrated in Figure 4, two electronic wires are applied and attached on the pulling clamp. The triangulation will be performed with the lengths measured by the Wire 1 and Wire 2 under different deflections. The UWB deflection will be mapped against the deflection estimated by electronic wires.

## 4. Full-Blade Time-Domain Measurement Results and Discussion

### 4.1. Measured Input Pulse Signals

**Figure 5:**
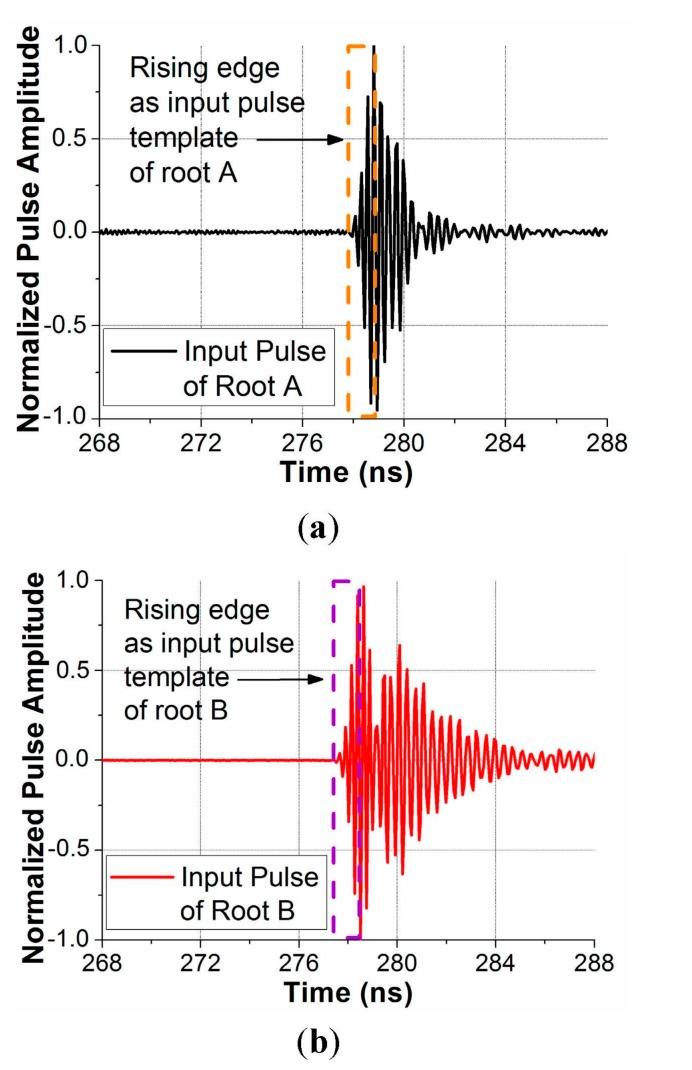
Normalized input (or transmitted) pulse waveforms obtained by the back to back measurements for (**a**) the root antenna A and (**b**) the root antenna B.

In order to obtain the waveform of the input signal rising edge, back-to-back measurements have been performed. In each measurement, the cable connected to one horn antenna and the cable connected to the tip antenna (see Figure 2) will be connected together through a step attenuator (−40 dB). The step attenuator can weaken the input signal amplitude to avoid clipping but without changing the waveform. In this way, we can find the signal waveform after passing through all system components (including waveguides, connectors, cables and so on) except for the antennas and channel. Figure 5 shows the normalized input pulse waveforms from the back to back measurements for the root antenna A and root antenna B. It is observed that the rising edges of the input pulses for the two root antennas are very similar, which will be used as the input pulse templates to compare with the output pulses. We also notice that the waveforms after the rising edge are a little different, which is mainly because the reflections at the connectors between two system components cannot always be the same. However, in this application, since we only compare the rising edge of input and output pulses, the difference after the rising edges will not affect the distance estimation accuracy.

### 4.2. Measured Output Pulse Signals

The blade has been pulled to static positions from −1 m deflection to 1.5 m deflection towards +y direction (see Figure 2) with 10 equal steps. At each position, the distances are measured with the UWB and laser range finder, and the deflection at the pulling clamp is estimated by the triangulation of the electronic wires. With the UWB system, approximate 50 samples are recorded at each position. Please note these 10 static positions are required by the laser range finder but not the UWB sensing system, since it is difficult to shoot the laser beam at the target when the blade is moving. Furthermore, since the root antennas and tip antenna are sharing the same coordinate system, the speed in z-axis direction is small. The motion of a blade on a real wind turbine will not lead to significant Doppler effects on the proposed UWB deflection sensing system.

**Figure 6:**
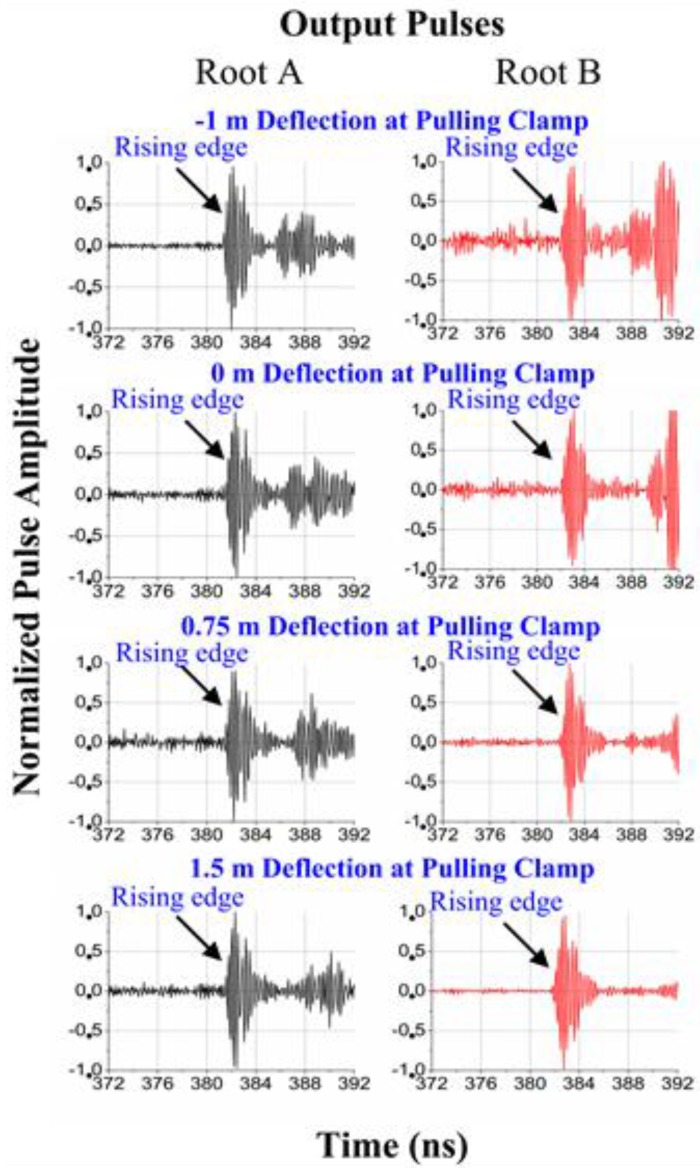
Normalized output pulse waveforms received by the root antenna A and the root antenna B.

The normalized output pulse waveforms received by the root antenna A and the root antenna B are given in Figure 6 for the different deflections. In each subfigure, the output waveform is normalized by the peak amplitude of the first pulse. In this application, the blade deflection is tracked by monitoring the location of the tip antenna. The tip antenna location is calculated by the triangulation with the two estimated tip-root antenna distances. The tip-root antenna distances are obtained by comparing the rising edge of the received and input pulse waveforms to estimate the time delay. It is observed in Figure 6 that the rising edges of the input pulses in Figure 5 are very similar to those of the output first pulses in Figure 6 under different deflections. It indicates the output first pulse fidelity is well kept under different deflections. Furthermore, the rising edge of the first pulse is clear and seldom interfered by the multipath effects under. Compared with the noise amplitudes in front of the rising edges, the first pulse amplitudes are always at least 14 dB stronger. Accurate and reliable distance estimations can be expected. Please note that in the original measured data, the received voltage in Figure 6 is not normalized. The voltages are normalized to facilitate comparison between the input and output pulse waveforms.

**Figure 7:**
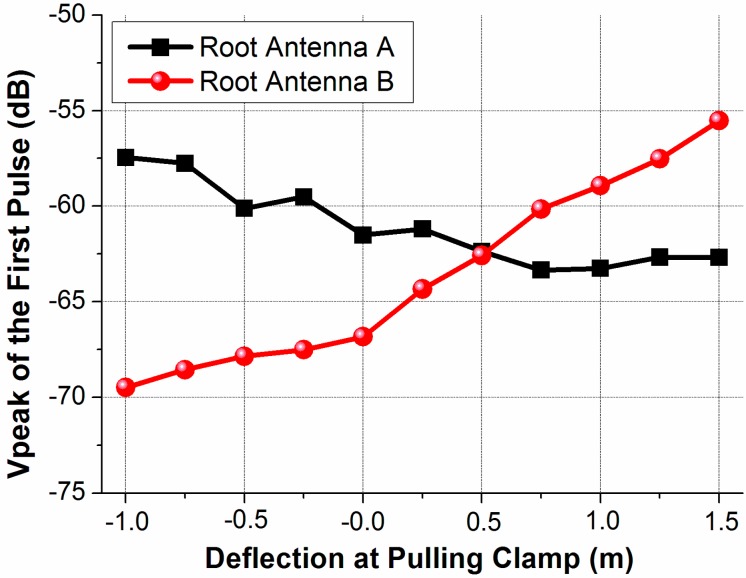
Peak voltages of the output first pulse under different deflections for the root antenna A and the root antenna B.

The peak voltages (or amplitudes) of the output first pulses are given under different deflections in Figure 7, which is normalized by the input pulse peak voltage. As the deflection changes, the peak voltages vary less than 6 dB and 15 dB for the root antenna A and the root antenna B, respectively. The lowest peak voltage of the root A occurs in the most important deflection range from 0 m to 1.5 m. However, with the selected height of the root antenna A off the blade root surface, the lowest peak voltage of the root antenna A is only 6 dB smaller than its highest value at −1 m deflection.

### 4.3. UWB Distance Estimation and UWB Blade Deflection Tracking

**Figure 8:**
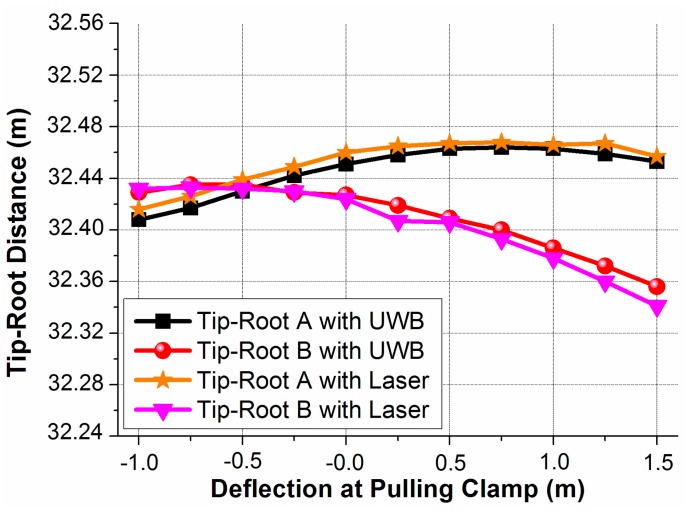
Comparison of the UWB and laser distance estimations.

The blade tip-root distance estimations with UWB radio and laser range finder are compared and shown in Figure 8. We can observe difference less than 1.5 cm from −1 m deflection (pre bending) to 1.5 m deflection. The resolution and accuracy of the signal processing algorithm may introduce some uncertainty. Part of the 1.5 cm difference is caused by this uncertainty. However, using a better signal processing algorithm or a wider spectrum bandwidth the resolution and accuracy can be further improved.

In Figure 4, the electronic wires are attached to the pulling clamp, 0.7 m closer to the blade tip than the tip antenna. Thus the deflection resulting from the wires will be offset compared to the deflection at the tip antenna position. Finite element method model of the blade is used to determine this relation. From our studies, this relationship is approximately linear and with constant of 0.918.

**Figure 9:**
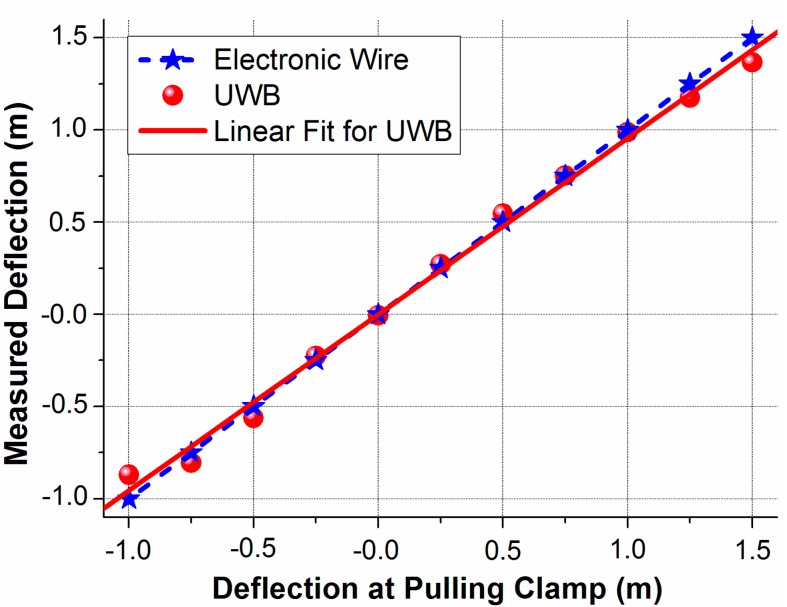
Comparison of the UWB and electronic wire deflection estimations.

The blade deflection at the tip antenna position is estimated using the triangulation of the two measured tip-root antenna distances with the UWB method in Figure 8. Then, this deflection is mapped from the tip antenna position to the pulling clamp position by dividing a factor of 0.918. The mapped UWB deflection is compared with the electronic wire deflection, which is shown in Figure 9. The difference between the two deflections is very small. To investigate the accuracy, we use “Y” and “X” to represent the “Measured Deflection” and “Deflection at Pulling Clamp” in Figure 9, respectively. For the electronic wire, it is expressed as Y = X, since both X and Y are directly measured by the wire from the camp. A linear fit is calculated for the mapped UWB deflection and expressed as Y = 0.95497 * X + 0.00358 with the coefficient of determination of 0.9944. By comparing the slope of the UWB deflection and electronic wire deflection, we get a correspondence within 4.5%. The maximum deviation and root mean squared error are 0.13344 m and 0.03911 m, respectively.

## 5. Conclusions

In this paper, we have introduced a new application of utilizing UWB technology for wind turbine blade deflection sensing. The detailed topology and challenges of this application have been provided. To prove this concept in the simplest case, full-blade time-domain measurements have been designed and carried out under different deflections. By properly selecting the root and tip antenna locations, UWB technology have successfully estimated the blade tip-root distance with the accuracy of 1.5 cm and the blade deflection with the error less than 4.5%.

This paper has realized the UWB radio link in the simplest case. However, there are still a number of future topics on this promising application. The following are some possible topics:

1. Design of an extremely low profile tip antenna placed outside a blade.
2. Investigation of UWB radio link for a tip antenna inside a blade.
3. Technologies of suppressing the interference and cancelling from multipath, reflections and surface wave for a tip antenna inside a blade.
4. Theoretical wireless channel modeling for a tip antenna inside and outside a blade.
5. Investigation of the blade deflection sensing with the UWB higher band or mm wave bands.
